# Supplementary material for: Local human movement patterns and land use impact exposure to zoonotic malaria in Malaysian Borneo
Source: eLife. 2019 Oct 22;8:e47602. doi: 10.7554/eLife.47602 (PMC6814363; doi:10.7554/eLife.47602)
Supplement: Supplementary file 1. [file elife-47602-supp1.docx]

**Supplementary file 1. Data sources for assessed spatial and environmental covariates**

| Covariate | Description | Spatial resolution | Source | Reference |
| --- | --- | --- | --- | --- |
| Land use type | Classified land use type as forest, agriculture, clearing and water | 30m | Derived from Landsat, described in Fornace et. al, *Plos Neglected Tropical Diseases*, 2018 | Landsat 8 Operational Land Imager. NASA EOSDIS Land Processes DAAC, USGS Earth Resources Observation and Science (EROS) Center. 2014. Available from: http://landsat.usgs.gov//index.php. |
| EVI | Monthly mean enhanced vegetation index (0-1) | 250m | MODIS Vegetation Indices | DAAC NL. MODIS/ Terra Vegetation Indices 16-Day L3 Global 250m Grid SIN V006. Sioux Falls, South Dakota: USGS Earth Resources Observation and Science (EROS) Center. |
| TWI | Topographic wetness index | 30m | Calculated from ASTER GDEM | Advanced Spaceborne Thermal Emission and Reflection Radiometer Global Digital Elevation Model (ASTER GDEM) Version 2 [Internet]. NASA EOSDIS Land Processes DAAC, USGS Earth Resources Observatoin and Science (EROS) Center. 2015. Available from: http://gdem.ersdac.jspacesystems.or.jp/ |
| Elevation | Meters above sea level (m) | 30m | ASTER GDEM |  |
| Slope | Degrees of slope incline (degree) | 30m | Calculated from ASTER GDEM |  |
| Aspect | Compass direction slope is facing (degree) | 30m | Calculated from ASTER GDEM |  |
| Distance to houses | Euclidean distance from nearest household, calculated from household GPS points (m) | 1m | Mapped during study using Garmin GPS, calculated in R |  |
| Distance to roads | Euclidean distance from nearest road, calculated from GPS tracks of roads (m) | 1m | Mapped during study using Garmin GPS, calculated in R |  |
| Population density | Estimated population per 100m for 2015 (adjusted for UN estimates) | 100m | WorldPop | Lloyd CT, Sorichetta A, Tatem AJ. High resolution global gridded data for use in population studies. Sci Data. 2017;4:170001. |
| Precipitation | Average monthly rainfall (mm/ day) | 0.25° | Tropical Rainfall Measuring Mission | Tropical Rainfall Measurement Mission Project (TRMM). Daily TRMM and other satellites precipitation product (3B42 V6 derived). In: DAAC) GSFCDAACG, editor. 2015. |
| Temperature | Average monthly temperature (°C) | - | Malaysian Meteorology Department |  |
