# Supplementary material for: Local human movement patterns and land use impact exposure to zoonotic malaria in Malaysian Borneo
Source: eLife. 2019 Oct 22;8:e47602. doi: 10.7554/eLife.47602 (PMC6814363; doi:10.7554/eLife.47602)
Supplement: Supplementary file 2. [file elife-47602-supp2.docx]

**Supplementary file 2. Data sources of mosquito biting data**

| Study | Study design | Sampling dates | Collection time | Data points | References |
| --- | --- | --- | --- | --- | --- |
| Longitudinal sampling | Longitudinal monthly sampling at sentinel sites in Matunggong and Limbuak | August 2013- December 2014 | 12 hours, 6pm – 6am | 82 | Chua TH, et al. Phylogenetic analysis of simian Plasmodium spp. infecting Anopheles balabacensis Baisas in Sabah, Malaysia. *PLoS Neg Trop Dis*. 2017;11(10):e0005991.  Wong ML, et al. Seasonal and Spatial Dynamics of the Primary Vector of Plasmodium knowlesi within a Major Transmission Focus in Sabah, Malaysia. *PLoS Neg Trop Dis*. 2015; 9(10):e0004135. |
| Case control | Sampling around houses of *P. knowlesi* cases and matched controls | February 2014 – July 2014 | 12 hours, 6pm – 6am | 34 | Manin BO, Ferguson HM, Vythilingam I, Fornace K, William T, Torr SJ, et al. Investigating the Contribution of Peri-domestic Transmission to Risk of Zoonotic Malaria Infection in Humans. *PLoS Neg Trop Dis*. 2016;10(10):e0005064. |
| Environmentally stratified sampling | Monthly sampling in randomly selected 100m^2^ grid cells, stratified by land cover | February 2015 – December 2015 | 6 hours, 6pm – 12am | 212 | Ng SH, Homathevi R, Chua TH. Mosquitoes of Kudat: species composition and their medical importance (Diptera: Culicidae). *Serangga*. 2016;21(2):149-62. |
