# Supplementary material for: Local human movement patterns and land use impact exposure to zoonotic malaria in Malaysian Borneo
Source: eLife. 2019 Oct 22;8:e47602. doi: 10.7554/eLife.47602 (PMC6814363; doi:10.7554/eLife.47602)
Supplement: Transparent reporting form [file elife-47602-transrepform.pdf]

eLIFE
1st Floor
24 Hills Road
Cambridge CB2 1JP, UK
P 01223 855340
W elifesciences.org
T @elife

eLife's transparent reporting form

We encourage authors to provide detailed information *within their submission* to facilitate the interpretation and replication of experiments. Authors can upload supporting documentation to indicate the use of appropriate reporting guidelines for health-related research (see EQUATOR Network), life science research (see the BioSharing Information Resource), or the ARRIVE guidelines for reporting work involving animal research. Where applicable, authors should refer to any relevant reporting standards documents in this form.

If you have any questions, please consult our Journal Policies and/or contact us: editorial@elifesciences.org.

Sample-size estimation

- You should state whether an appropriate sample size was computed when the study was being designed
- You should state the statistical method of sample size computation and any required assumptions
- If no explicit power analysis was used, you should describe how you decided what sample (replicate) size (number) to use

Please outline where this information can be found within the submission (e.g., sections or figure legends), or explain why this information doesn't apply to your submission:

No sample size was calculated for this work as these were rolling cross sectional surveys designed to be representative of the study areas. The minimum number and criteria for inclusion are described in Methods, under GPS tracking survey (Lines 142- 146).

Replicates

- You should report how often each experiment was performed
- You should include a definition of biological versus technical replication
- The data obtained should be provided and sufficient information should be provided to indicate the number of independent biological and/or technical replicates
- If you encountered any outliers, you should describe how these were handled
- Criteria for exclusion/inclusion of data should be clearly stated
- High-throughput sequence data should be uploaded before submission, with a private link for reviewers provided (these are available from both GEO and ArrayExpress)

Please outline where this information can be found within the submission (e.g., sections or figure legends), or explain why this information doesn't apply to your submission:

This study design (rolling cross sectional surveys) did not include replicates and the sampling procedures are described in the Methods under GPS tracking survey. For mosquito biting data used, the full details on the number of replicates and number of locations and nights are included in the Results and Supplementary file 1B, with full data available through the referenced publications.

eLife Sciences Publications, Ltd is a limited liability non-profit non-stock corporation incorporated in the State of Delaware, USA, with company number 5030732, and is registered in the UK with company number FC030576 and branch number BR015634 at the address 1st Floor, 24 Hills Road, Cambridge CB2 1JP | August 2014

Statistical reporting

- Statistical analysis methods should be described and justified
- Raw data should be presented in figures whenever informative to do so (typically when N per group is less than 10)
- For each experiment, you should identify the statistical tests used, exact values of N, definitions of center, methods of multiple test correction, and dispersion and precision measures (e.g., mean, median, SD, SEM, confidence intervals; and, for the major substantive results, a measure of effect size (e.g., Pearson's r, Cohen's d)
- Report exact p-values wherever possible alongside the summary statistics and 95% confidence intervals. These should be reported for all key questions and not only when the p-value is less than 0.05.

Please outline where this information can be found within the submission (e.g., sections or figure legends), or explain why this information doesn't apply to your submission:

- Overview of all statistical methods used, Supplementary Figure 1
- Biased random bridges, description of methodology (Methods, Human Space Use); number analyzed and duration (Results, Lines 279 - 286); home range estimates with IQR (Table 1)
- Resource utilization models, data sources (Methods, Supplementary File 1A), description of methodology (Methods, Resource utilization functions); results and model fit (Results, Table 2, Figure 3 B)
- Mosquito biting models, data used (Figure 2 A and B, Supplementary File 1A and B, Figure S3 A), model development (Methods), results (Tables 3 and 4, Figure 3 C)
- Calculation of exposure, methods (Methods), Results (Results, Figure 2 C and D, Figure 3 D)

(For large datasets, or papers with a very large number of statistical tests, you may upload a single table file with tests, Ns, etc., with reference to sections in the manuscript.)

Group allocation

- Indicate how samples were allocated into experimental groups (in the case of clinical studies, please specify allocation to treatment method); if randomization was used, please also state if restricted randomization was applied
- Indicate if masking was used during group allocation, data collection and/or data analysis

Please outline where this information can be found within the submission (e.g., sections or figure legends), or explain why this information doesn't apply to your submission:

This information does not apply to this submission as no experimental groups were included.

Additional data files ("source data")

- We encourage you to upload relevant additional data files, such as numerical data that are represented as a graph in a figure, or as a summary table
- Where provided, these should be in the most useful format, and they can be uploaded as "Source data" files linked to a main figure or table
- Include model definition files including the full list of parameters used
- Include code used for data analysis (e.g., R, MatLab)
- Avoid stating that data files are "available upon request"

Please indicate the figures or tables for which source data files have been provided:

Full details of all models are included in the main text and SI. The SI also contains details of all publicly available datasets of environmental variables used for this analysis and publications including all entomological data.

The individual GPS tracking data cannot be made publicly available as due to ethical constraints around the inclusion of identifying information. Scripts to perform this analysis in R are included as supplementary information and will be made available on https://github.com/kfornace
